# Which-way identification by an asymmetrical double-slit experiment with monochromatic photons

**DOI:** 10.1038/s41598-022-07662-x

**Published:** 2022-03-08

**Authors:** Thuan Vo Van, Vinh Vu Duc

**Affiliations:** 1 Institute of Theoretical and Applied Research (ITAR), Duy Tan University, Hanoi, 100000 Vietnam; 2 Faculty of Natural Sciences, Duy Tan University, Da Nang, 550000 Vietnam; 3 Vietnam Atomic Energy Institute (VINATOM), 59 Ly Thuong Kiet street, Hoan Kiem district, Hanoi, Vietnam

**Keywords:** Quantum optics, Single photons and quantum effects

## Abstract

Recently, a laser beam asymmetrical double-slit experiment was proposed and performed, concerning ontological physical reality in quantum mechanics, under an assumption of single-photon interference. In the present study, by controlling better for saturation effects and upgrading the slit’s shape, we succeed in producing new interference samples with acceptable quality. Applying almost the same geometrical set-up, the present experiment makes the ”which-way” identification with higher experimental confidence. In the results, the ontological which-way effect observed in our recent experiment is well reconfirmed without any additional measurement of relative integral intensity.

Following the classical Young interference, the double-slit experiments with single photons^1,2^ served as an illustration of wave-particle duality in quantum mechanics (QM), then similar experiments with electrons^3–5^ were performed. Feynman considered that a double-slit experiment with single electrons using a movable mask for closing or opening one of the slits would serve as a key test of the fundamental problem of physical reality in quantum mechanics^6^. Only recently, based on state-of-the-art electronic microscopy, the so-called Young-Feynman ”thought” double-slit experiments have finally become real^7,8^, which seems to confirm the incompatibility of wave and particle features of a quantum substance following the principle of complementarity. Regarding de Broglie-Bohm theory with hidden parameters^9–11^, experimental verification of Bell inequality^12,13^ in the early 1980s denied the local physical reality. A class of non-local reality defined by Leggett inequality has also been rejected^14,15^. This does not mean the debate for physical reality in quantum mechanics is over. Indeed, the door is still open for some classes of physical reality with non-locality, such as the theories with extra-dimensions (see, e.g.,^16^). Recently, following the idea of weak measurements^17^, Steinberg et al.^18^ determined statistically averaged Bohmian trajectories, which supports the non-locality of the physical reality^19^. The latter does not mean any violation of either statistical principle or Heisenberg indeterminism^20^. From another perspective, asymmetrical double-slit experiments with different slit-width for heavy particle beam was proposed^21^ and a recent asymmetrical double-slit experiment with electrons demonstrates a possibility of identification of the ”which-way” physical reality, which seems to identify qualitatively the two different diffraction patterns with interference from the two slits in a pre-Fraunhofer condition^22^. Considering the importance of this problem, we proposed and carried out another asymmetrical double-slit experiment with monochromatic photons in the far-field condition^23^ which succeeded not only in a qualitative identification of interference patterns regarding different slits but also revealed several true ”which-way” signals without disturbance of the photon beam in some locations of missing interference orders. In the present study, we report on the results of a similar experiment with lower photon intensity in the vicinity of the main minima, for upgrading and reconfirmation of the previous which-way phenomenon by a new design of the asymmetrical double-slits.

## Results

### Conservation of missing interference orders in the diffraction pattern $D_1^{int}$

Quantum physics links the monochromatic wave to a light particle, the photon. Following the method in^23^, below-briefed in session 4 for the single-photon condition the size of a corresponding diffraction pattern indicates what slit the photon assemble passes through. In this consideration, the photon is a discrete intact quantum particle, based on its objective ontological physical reality. Since the self-interference of a single-particle has been firmly observed^2,5^, one possible method of interpretation for this phenomenon may follow the de Broglie-Bohm theory^9–11^ in which the accompanying pilot-wave, passing both slits, drives the particle passing through a single slit, moving along a Bohmian trajectory toward to a certain interference order. In a summation spectrum following Equation (9) different sizes of the two mixing patterns $D_m^{int}$ make their interference-missing orders localize in different positions. In the results, an interference fringe of the first constituent pattern can stand on an empty missing order of the second pattern, which allows identification of the first slit as the only path of the photons coming to this fringe. Truly so, in a typical laser time window ($\sim 10^{-15} sec.$) no concurrent monochromatic photon component from the second slit can occur in these special locations.

In general, in the self-interference of single-photons, interference orders of both constituent diffraction patterns in (9) coincide, which means their corresponding interference fringes are miscible and inseparable. But it is never true in the interference missing-orders, where the contribution comes from a certain diffraction pattern uniquely. This assumption serves as a basis for the identification of the path of single-photons by a post-analysis of their asymmetrical double-slit interference spectrum without touching the laser beam during an experimental sampling. Probably, a causal dBB-like interpretation ensures that the self-interference of a single photon^2,5^ is a unique ontological effect of the quantum substance. In the present experiment, the proposed conservation law of the interference missing-orders and of the sizes of diffraction patterns should be reconfirmed.

At variance with our recent experiment^23^, due to enlarged spacing distance between the two slits $d\ge 1.$ mm, the six laser beam directions (instead of five) are newly fixed at six angles relabeled as $\alpha _1\div \alpha _6$ as can be seen in the layout, “Experimental set-up” subsection. The optic physical quantities are defined in “Theoretical ontological concept” subsection.

**Figure 1:**
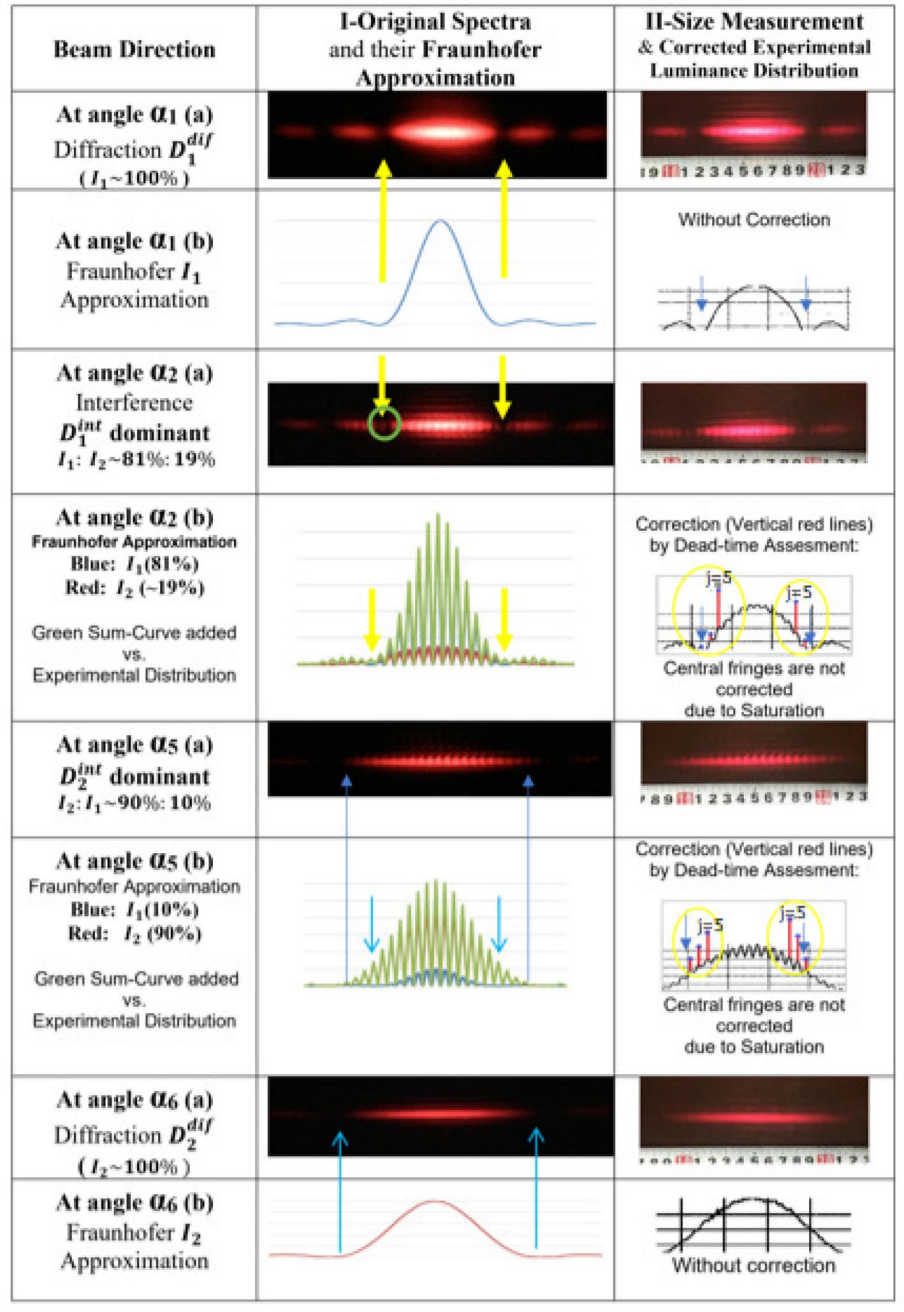
”Which-way” identification by sizes of the central bands $D_m^{int}$: Shorter spectrum regards $I_1$-photons passing through the wide-slit. $I_1$-photons are absent in the first main minima (indicated by yellow arrows) of $D_1^{dif}$ at $\alpha _1$. Contributions $I_1$ and $I_2$ are integrated between their corresponding main minima at $x_i=\pm x_{1}$ (blue arrows). Experimental luminance distributions in Column (II) for directions at $\alpha _2$ and $\alpha _5$ after correction of interference fringes with $j=5\div 7$ (red lines in yellow ellipses) fit well the Fraunhofer approximation in the vicinity of the main minima.

**Figure 2:**
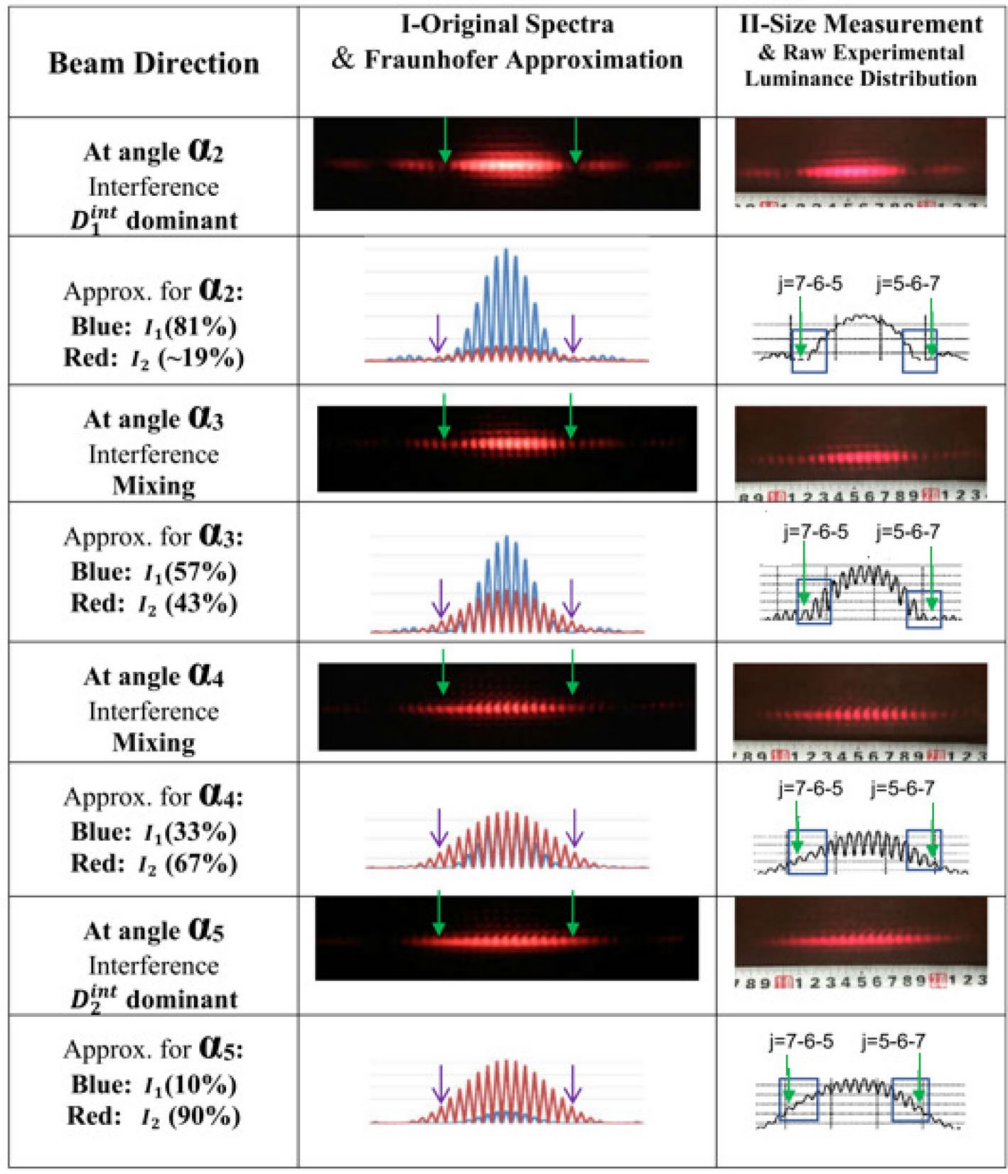
True ”which-way” signals: Direct observation of pure interference fringes with luminance *I*(*min*.) of $I_2$-photons passing through the narrow-slit (indicated by green arrows in spectra and Violet arrows in corresponding approximation curves). They are seen also in rectangular boxes with increasing luminance $I(min.)=I(j=7)$ (indicated by green arrows) together with their neighboring fringes with luminance $I(j=6)$ and $I(j=5)$ when the laser beam changes its direction from $\alpha _2$ to $\alpha _5$.

Spectra measured and photographed at all six angles are posted in Figs. 1 and 2 for analysis. In Fig. 1 a correlation between the beam direction and the spectrum size is emphasized. In Directions at $\alpha _1$ and $\alpha _2$ a short length of the diffraction patterns $D_1^{dif}$ and $D_1^{int}$ regards the wide slit, while in Directions at $\alpha _5$ and $\alpha _6$ a long length of the patterns $D_2^{int}$ and $D_2^{dif}$ is induced by the narrow slit, which is consistent with Equations (5) and (6). The experimental sizes of spectra $D_m^{int},\ (m=1,2)$ and the order spacing of interference fringes $\Delta F$, and numbers of fringes in interference patterns $n_m$ vs. their calculated quantities are presented in Table 1.

**Table 1:** Sizes of spectra and interference fringes

Quantity	Calculation	Experiment
$D_1^{int}$, cm	6.9	$7.7 \pm 0.5$
$D_2^{int}$, cm	12.3	$14.0\pm 0.8$
$\Delta F$, cm	0.54	$0.60 \pm 0.05$
$n_1$	$[12.9]\equiv 13$	$13.0 \pm 0.5$
$n_2$	$[22.9]\equiv 23$	$23.0 \pm 1.0$

There the interference order spacing $\Delta F$ and the experimental lengths $D_m^{int}$ are measured (in cm) between the two first main minima of the central bands in original 2D-images, presented in Columns(II), Figs. 1 and 2. Because the number of fringes $n_m$ in each central band is increased by approximately double compared to the previous experiment, the same initial photon intensity is now distributed more softly to each fringe, which constrains the optical density, thus better controlling the saturation effect. In particular, the newly designed double-slit conserves the photon rate closer to the true one in the vicinity of the main diffraction minima. In Table 1 for the slit-widths $\{b_1,\ b_2\}$ measured with finite uncertainties, it is found that the experimental data are consistent with their quantities calculated by Formulas (3), (5), (6) and (7), which serves as an optimal quality control of the presented measurements. As a result, the numbers $n_1=13$ and $n_2=23$ will serve for correctly ordering and spacing of interference fringes in their corresponding diffraction bands. This ensures searching for pure minimum locations at $x_i=\pm x_1$ of the diffraction pattern $D_1^{dif}$, simultaneously, being the missing orders of its corresponding central pattern with interference $D_1^{int}$. In comparison with the intensity distribution in^23^ the softer luminance of interference fringes allows for enlarging the useful vicinity of the main minima, at least for two fringes with $j=6$ and $j=5$ neighboring to the missing orders in $j=7$.

In our methodological study^23^, a $99\%$-confidence of the experimental purity (i.e. cleanness from the $I_1$-photons) is recorded in minimum locations $x_i=\pm x_1$. Similarly, in the present Fig. 1-Column (I) the two pure locations are shown by the arrows in the two first main minima of the diffraction pattern $D_1^{dif}$ at $\alpha _1$ or in the corresponding missing interference orders of the pattern $D_1^{int}$ at $\alpha _2$, which confirms that in the minimum gaps there is no signal of $I_1$-photons passing through the wide slit except a weak dot like a signal of $I_2$ or a common mixing background $\Delta I_1$ (this signal can be seen in the green circle). In the same manner, the two first minima are proven clean enough from any signal of $I_1$-photons with a constraint of the background $\Delta I_1\le 1.\%$. In addition, Column (II) displayed the experimental luminance distributions, obtained from the 2D-images of interference patterns $D_1^{int}$ and $D_2^{int}$ performed by the ISee-scanning software, having been introduced by the International Atomic Energy Agency (IAEA). The ISee-code warrants a routine statistical fluctuation of roughly $(5.\div 10.)\%$ in the vicinities of the main minima of each experimental luminance distribution. The dead-time corrected distributions by Formula (10) being consistent with their corresponding Fraunhofer approximations in Column (I) would prove the conservation of missing interference orders in the diffraction pattern $D_1^{int}$ in the asymmetrical double-slit mixing spectra. It is essential as for the primary objective of the present subsection that a missing interference order in each of the first minima reserves an $I_1$-empty spacing $\Delta F$ exactly in the first main minima $x_i=\pm x_1$ which can serve for the accommodation of another valid interference fringe with luminance $I(min.)\equiv I(j=7)$ originated from $I_2$-photons, which will be reported bellow. In principle, this conservation law can be extended to other main minima of both interference patterns $D_1^{int}$ and $D_2^{int}$.

### Identification of experimental evidence of the which-way effect

In Fig. 1 one can notice a reminiscence in the size and in the shape of the interference pattern $D_1^{int}$ at $\alpha _2$ with the $I_1$-regarding diffraction pattern $D_1^{dif}$ at $\alpha _1$, and a similar reminiscence of the interference pattern $D_2^{int}$ at $\alpha _5$ with the $I_2$-diffraction pattern $D_2^{dif}$ at $\alpha _6$, correspondingly. Therefore, a qualitative which-way effect can be seen in interference patterns not only in the post-Frenel near-field condition as in the experiment with electrons^22^, but also in the Fraunhofer far-field condition. Generally, the correlations of a pattern length or a relative integral intensity $I_1/I_2$ with a corresponding laser beam direction serve as strong arguments for a qualitative ”which-way” identification.

However, an assessment of the contribution of each component $I_1$ or $I_2$ cannot identify their pure ontological ”which-way”, because, one can never isolate the interference mixing photons passing through one or another slit. Any attempt to label a given slit of a photon path will fail to conserve interference fringes, as happened in the recent double-slit experiments with a Feynman condition^7,8^.

Because of the fixed widths of both slits $\{b_1, b_2\}$, a truly constant distance *d* makes the size $\Delta F$ of an interference fringe identical in all spectra. As a result, one can expect that the overlapped interference fringes of both constituent patterns are well-coincident. Therefore, it is enough to fit the brightest central fringes of different patterns $D_m^{int}$ for comparison, then the location of the two first minima of the diffraction pattern $D_1^{int}$ are well determined at $x_i=\pm x_1$. Correspondingly, all interference fringes regarding the four laser directions from $\alpha _2$ to $\alpha _5$ are presented in Fig. 2 together with their original experimental luminance distributions (produced by the ISee code). After removing a stationary background in the laboratory environment, these distributions are recovered partially by electronic pixel dead-time assessment, which succeeds in correcting the low-intensity fringes, particularly, for those fringes near to the minima $x_i=\pm x_1$ with interference orders $j=6$ and $j=5$ (but not for fringes in the center due to saturation). The corrected distributions by Formula (10) being consistent with their corresponding Fraunhofer approximations in Column (I) will serve as a basis for the ontological independence of $D_1^{int}$ and $D_2^{int}$ following Summation (9).

In fact, in Fig. 2, an interference fringe appearing in each missing order in the next spectra becomes brighter when the laser reorients its direction from the direction at $\alpha _2$, through $\alpha _3$ and $\alpha _4$ to the direction at $\alpha _5$. Images of those two selected fringes are indicated by green arrows in Fig. 2-Column (I), which fill the minima from almost empty gaps in $D_1^{int}$ (at $\alpha _2$) to full-up locations in $D_2^{int}$ (at $\alpha _5$), thus the fringes finally reach a standard size $\Delta F$ in the pattern $D_2^{int}$ in the direction at $\alpha _5$. This leads to quantitative analysis for ontological identification of the photon’s path.

## Discussion

A post-analysis of experimental luminance *I*(*j*) of the interference fringes in the order with $j=7$ has been carried out to prove their original which-way. Throughout our research instead of absolute quantities *I*(*j*), the relative luminance $R[j/(j-1)]$ of neighboring interference fringes is applied, which facilitates overcoming some extra sources of systematic errors. For data processing, the triples of neighbor fringes with $j=5,6,7$ are selected from the six ISee spectra in Figs. 1 and 2. The experimental area of each $j-$fringe taken as the original sampling $N_j$ determines its statistical error. Following a standard procedure, a fringe’s area is calculated, taking into account some adjustment due to partial overlapping of neighbor fringes, caused by a certain incoherence of the laser beam and by elastic scatterings. The fringe areas of both sides left and right, are then averaged for compensation of unexpected asymmetry. Furthermore, all statistical samples $N_j$ are reduced by the renormalization factor $R_N=9$ to meet the dead-time correction approximation with $\tau _d=0.012$, following Equation (10). In the results, the original luminance of three interference fringes in the vicinity of the first main minima and their corrected quantities are presented in Table 2 together with their main sources of uncertainties, where $I_{obs}(j)=N_j/R_N$ have been renormalized. The statistical errors routinely equal to $5\%\div 10\%$ except for the direction at $\alpha _1$ are shown for $I_{obs}(j)$.

**Table 2:** Experimental luminance of fringes in the vicinity of the first main minima: the observation ( from Column (II), Fig. 2) and the dead-time corrected quantities (by correction factor $\tau _d=0.012$). Uncertainties of $I_{obs}(j)$ are statistical errors, while uncertainties of the corrected luminance $I_{true}(j)$ consist of statistical and three kinds of systematical errors by $\Delta \tau _d$, Multi-photon interference and slit microstructure defects.

Direction at angles	$\alpha _1$	$\alpha _2$	$\alpha _3$	$\alpha _4$	$\alpha _5$	$\alpha _6$
$I_{obs}(7)$	$0.65\pm 0.3$	$11.0\pm 1.2$	$15.2\pm 1.3$	$20.6\pm 1.6$	$27.1\pm 1.8$	$18.2\pm 1.5$
$I_{obs}(6)$	$13.4\pm 1.3$	$20.7\pm 1.5$	$23.5\pm 1.7$	$27.9\pm 1.8$	$35.1\pm 2.0$	$23.4\pm 1.7$
$I_{obs}(5)$	$47.7\pm 2.3$	$43.2\pm 2.2$	$38.1\pm 2.1$	$35.6\pm 2.0$	$41.1\pm 2.2$	$27.3\pm 1.8$
$I_{true}(7)$	$0.66\pm 0.6$	$12.6\pm 1.4$	$19.0\pm 2.5$	$27.6\pm 3.8$	$40.1\pm 4.2$	$23.2\pm 2.3$
$I_{true}(6)$	$16.0\pm 3.6$	$29.0\pm 2.9$	$35.2\pm 4.8$	$42.0\pm 6.0$	$61.0\pm 7.6$	$31.5\pm 3.1$
$I_{true}(5)$	$112.\pm 22.$	$92.4\pm 14.9$	$73.9\pm 11.1$	$62.1\pm 9.3$	$81.3\pm 12.1$	$40.5\pm 4.3$

Instead of this, Table 2 demonstrates the experimental errors of $I_{true}(j)$ combined statistical errors with systematical uncertainties. The latter comes from three kinds of competitive systematical errors. Firstly, an unfitness of the empirical dead-time correction factor ($\tau _d=0.012$), estimated $< 20\%$ for $I_{obs}\le 50$ following Equation (10) which impacts more the data of $I_{true}(5)$. Secondly, a possible multi-photon interference contribution $\le \delta _M(I_1,I_2)$ is estimated as significant, roughly $16\%\div 17\%$ in the intermediate directions at $\alpha _3$ and $\alpha _4$, but would be less in the vicinity of the main minima. Finally, a left-right asymmetry caused by an uncontrolled deformation of the slits, routinely $\le 5\%$, but maximally estimated for *R*(7/6) reaching a $97\%$-value due to a poor statistics in the direction at $\alpha _1$ and roughly equal to $13\%$ at $\alpha _2$.

The luminance of each of the two selected interference fringes *I*(7) in the first interference missing orders of the pattern $D_1^{int}$ is analyzed by intercomparison with its neighboring fringes, with $j=6$ or $j=5$ (they are all three indicated in a rectangular box in Column (II), Fig. 2). At variance with *I*(7), luminance *I*(6) or *I*(5) is mixed from both $D_1^{int}$ and $D_2^{int}$. In combination the above-mentioned uncertainties cause experimental errors $\Delta R_Y\%$ of the relative luminance $R(7/6)=I_{true}(min.)/I_{true}(6)$ from $14\%$ to $20\%$, except for the direction at $\alpha _1$ due to poor statistics and a possible relatively large left-right asymmetry of $I_{obs}(7)$ in the spectrum. Correspondingly, the combined uncertainty leads to the experimental errors $\Delta R_Z\%$ of $R(6/5)=I_{true}(6)/I_{true}(5)$ from $14\%$ to $30\%$.

**Figure 3:**
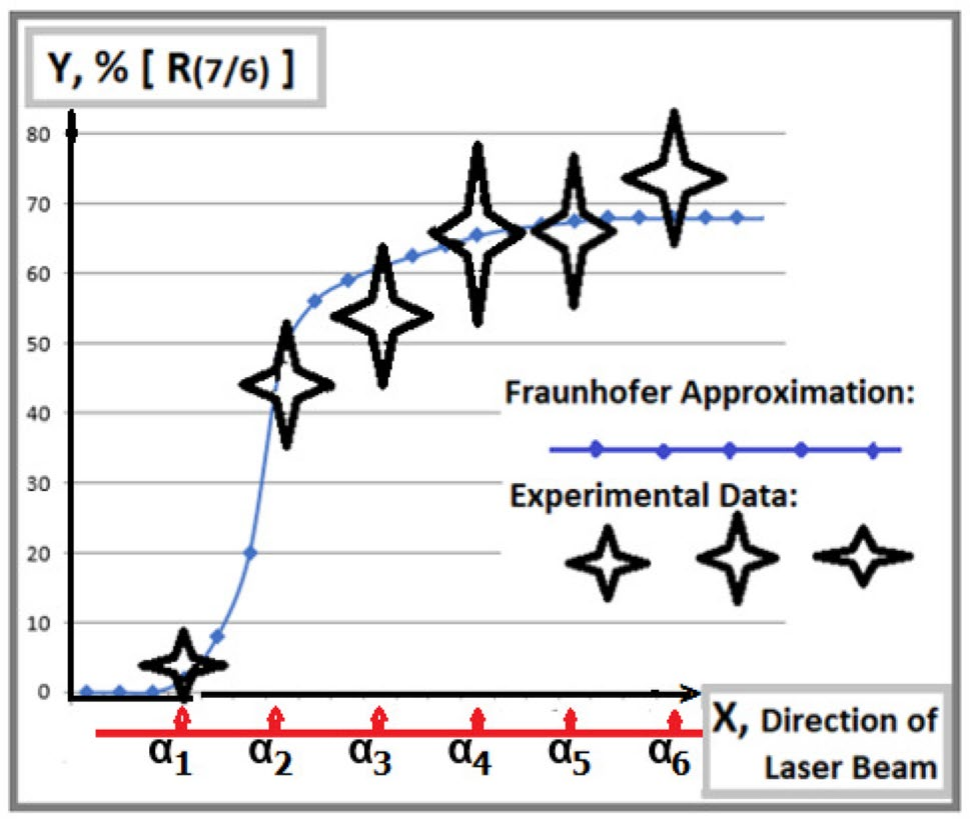
A relative luminance of fringes in $x=\pm x_1$ to their neighbors vice-versa horizontal directions of the laser beam: A strong correlation of Experimental ratio $R(7/6)=I(min.)/I(6)$ with Fraunhofer approximation curve is consistent with the increasing contribution of $I_2$-photons while going against the decreasing $I_1$.

After restoration by the dead-time correction, the relative luminance *R*(7/6) is posted in Fig. 3 in comparison with the theoretical ratio calculated by the Fraunhofer approximation in Column (I), Fig. 2.

**Figure 4:**
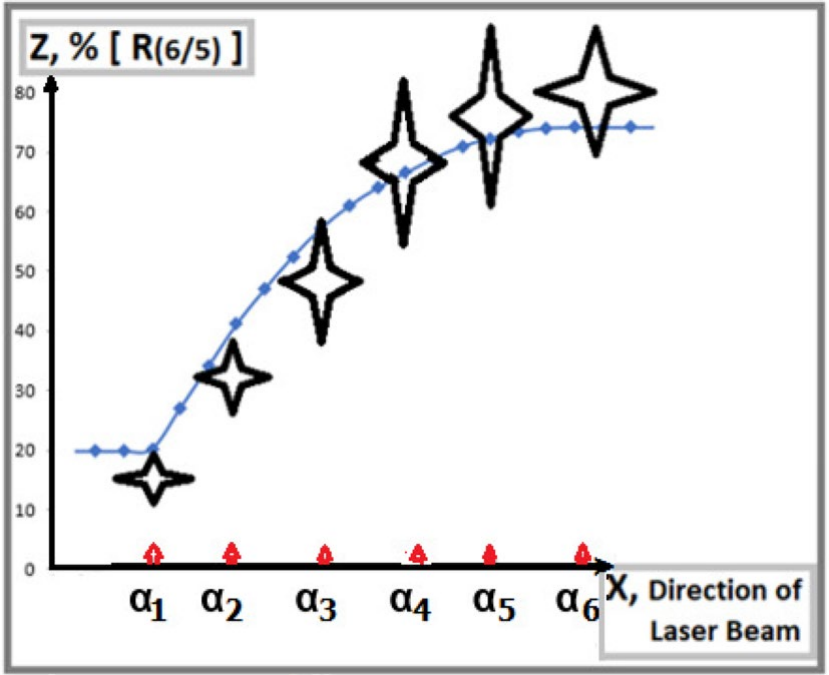
A relative luminance of neighboring interference fringes in the vicinity of the main minima vise-versa horizontal directions of a laser beam: A correlation of experimental ratio $R(6/5)=I(6)/I(5)$ with the Fraunhofer approximation curve is also consistent with the increasing contribution of $I_2$-photons.

Based on a relatively low optical density, a similar analysis can be extended to the relative luminance *R*(6/5) for comparison with the same corresponding theoretical Fraunhofer curves in Column (I), Fig. 2, which is presented additionally in Fig. 4.

In both figures Figs. 3 and 4, there is also significant uncertainty $\Delta X$ in fixing a laser beam direction in horizontal orientation because of an empirical extrapolation of relative integral intensity $I_1/I_2$ based on the old data presented in Fig. 7, “Experimental set-up” subsection. For a conservative assessment, the present $\Delta X$ increases by $\sqrt{2}$ times compared to the old $\Delta X$. Subsequently, for convenience, all experimental data are presented by four-wing stars, containing experimental errors along the ordinate and uncertainties $\Delta X$ along the abscissa.

At variance with the graphical presentation of *R*(7/6) in Fig. 3, the experimental data of *R*(6/5) in Fig. 4 shows a slight systematical declination with the theoretical curve, which would originate from some unfitness of the empirical dead-time factor and/or from microscopic deformation of each slit, in particular, in the laser beam directions at $\alpha _1$ and $\alpha _2$. Consequently, the correlations of both experimental sets *R*(7/6) and *R*(6/5) with their theoretical approximation curves are presented within the total combined errors, not only within their statistical fluctuation. In the results, they consistently confirm a quantitative correlation of the experimental luminance $I_{true}(7)$ with the increasing tendency of $I_2$-photons while against the decreasing tendency of $I_1$-photons in Fraunhofer approximation curves following Distribution (9).

**Figure 5:**
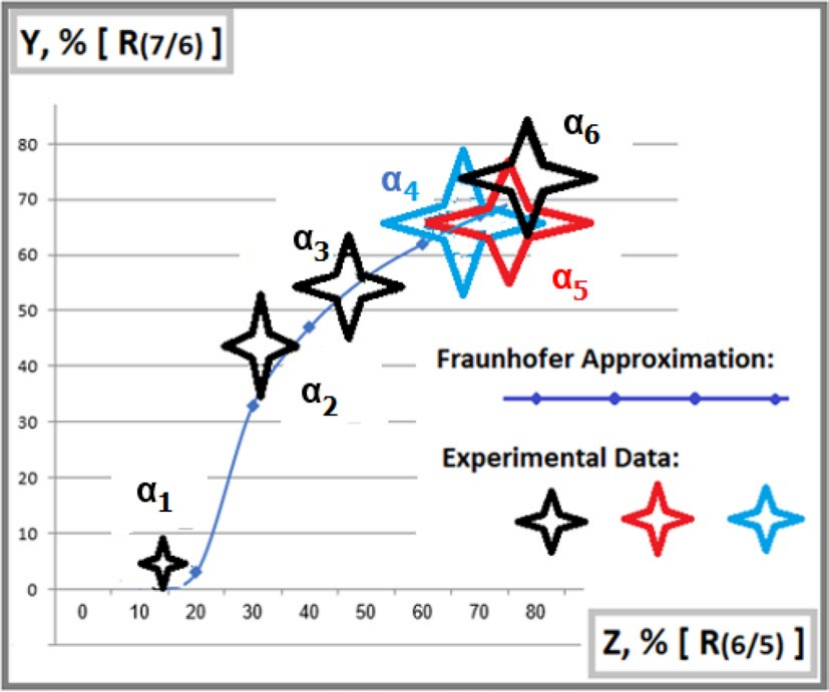
A correlation of Experimental ratio $R(7/6)=I(7)/I(6)$ with another Experimental ratio $R(6/5)=I(6)/I(5)$ is consistent with the expectation from the Fraunhofer approximation without any additional measurements of the relative integral intensity $I_1/I_2$ as a function of the laser beam directions at angles $\alpha _l$.

In the final step, to avoid any systematical uncertainties $\Delta X$ in fixing each laser beam direction along axis X by an empirical extrapolation of the relative integral intensity $I_1/I_2$ from the data in Fig. 7, let us consider Figs. 3 and 4 as two projections on XY- and XZ planes of a 3D-cartesian graphical presentation. Consequently, the third YZ plane contains a projected theoretical Fraunhofer approximation curve of a correlation between *R*(7/6) and *R*(6/5) and their corresponding experimental data as it is shown in Fig. 5. Following the new presentation, the research methodology is upgraded radically, because it doesn’t need any additional supporting measurement of separate diffraction patterns $D_m^{dif}$ in each laser beam direction by using a mobile mask in the Feynman condition. The only kind of measurement is to carry out sampling of a collection of interference patterns $D_m^{int}$ with any relative integral intensities $I_1/I_2$, which offer the correlated pairs of relative luminance, such as *R*(7/6) and *R*(6/5), like the experimental records presented in Fig. 5. There the reference theoretical curve following Summation (9) is simulated by varying a parameter $R(I_1/I_2)\sim I_0(1)/I_0(2)$. Recalling that the self-interference of single photons may be based on the hypothesis of their ontological feature following a de Broglie-Bohm-like interpretation in which a unique source of interference is the pilot-wave. Really, if there some quasi-elastic processes occur, e.g. Rayleigh scattering or Doppler shift by air molecules, the fringes even though enlarging, are conserved for interference counting. However, other inelastic channels, based on high-order Feynman diagrams would lead to decay of the single photons into smaller portions, passing through both slits, equivalent to classical incoherent waves, which could increase only the environmental background. Therefore, classical waves cannot contribute to the interference of single photons in the summation spectrum. From another perspective, for high-intensity beams, a large contribution of multi-photon interference would also approach classical optics. Nevertheless, an ordinary double-slit experiment with classical waves would be meaningless for the which-way identification. Due to a large deviation from the distribution described by the single-photon-based summation (9), now the two mixed components of its interference spectrum become no more independent. For this reason, the multi-photon interference cannot reveal any ontological feature, even in the former interference-missing orders in the main minima of constituent diffraction patterns.

Based on Table 2, the combined uncertainties of the final relative luminance quantities are generally higher than their sole routine statistical errors by a factor of roughly $1.5 \div 2.4$. Therefore, in Fig. 5 one finds a significant correlation of experimental data with the reference curve within experimental errors, which reconfirms the conclusion in the recent experiment^23^, that the newly appeared interference fringes in the missing interference orders with $j=7$ of $D_1^{int}$ are naturally assigned to $I_2$-photons passing through the narrow slit, as their unique path, but not to $I_1$-photons. In such an experimental sample, one can observe steadily the two interference fringes of the $I_2$-photons in $x_i=\pm x_1$ without any disturbance of the laser beam. This phenomenon implies a pure ontological ”which- way” identification in a summation interference spectrum, which confirms an observation of both the path and the momentum of the photons in the same measurement. This result seems to agree with causal interpretations of quantum mechanics, like the dBB-theory^9–11^, leading to the motion of microscopic particles along Bohmian trajectories^17–19^.

## Conclusions

The new experiment performed with several advantages can offer more information, not only on the variation of their relative luminance as a function depending on the laser beam direction but also of the experimental correlation between the two different relative luminance quantities *R*(7/6) and *R*(6/5) being consistent with the ontological-based Fraunhofer curve. The latter can avoid any systematical uncertainties which would occur in the assessment of relative integral intensity $I_1/I_2$ in a Feynman condition as in the previous experiment. In the results, the present double-slit experiment with monochromatic photons well reconfirms our recent asymmetrical double-slit observation. With higher experimental confidence, the new design of the double-slits used offers new evidence of the which-way identification of the wave-particle duality, which implies a possibility of simultaneous measurement of the path and the momentum of the photon in the same experiment. For multiple perspectives, it is desirable to carry out similar asymmetrical double-slit experiments with both single electrons and single photons for adequate and direct verification of the self-interference of a microscopic quantum substance.

## Method

### Theoretical ontological concept

A geometrical arrangement of the present experiment, being similar as reported in^23^ satisfies three conditions: i/ the asymmetrical double-slits; ii/ the single-photon approximation, and iii/ the Fraunhofer far-field. The following selected theoretical elements of classical optics would be extended to describe the hypothesis of the quantum pilot-waves^9–11^, governing ontological subjects, i.e. the single photons in the proposed experiment. In the far-field condition (the Fresnel number $N_{Fr}\approx 10^{-3} \ll 1.$), the Fraunhofer approximation of intensity distribution reads:

1 $$\begin{aligned} I_m(x)dx= |\psi _m(x,t)|^2dx \equiv I_0(m)|U_m(x)|^2dx =I_0(m)\left[ \frac{\sin u_m}{u_m}\right] ^2dx, \end{aligned}$$

where $I_0(m)=A_{0,m}^2$ is relative luminance maximum in the center ($x=0.$) of *m*-component. The phase $u_m(x)$ simply reads:

2 $$\begin{aligned} \ u_m(x)=\frac{\pi b_m.x}{L.\lambda }, \end{aligned}$$

where *L*, a distance from the slit-diaphragm to the screen; $\lambda$, wavelength; $b_m$ is slit-width labeled by $m=\{1,2\}$. In a traditional double-slit experiment, symmetrical slits are used to generate interference fringes. The size $\Delta F$ of each fringe is inversely proportional to the distance *d* between the two slits as follows:

3 $$\begin{aligned} \Delta F=L\frac{\lambda }{d}. \end{aligned}$$

An *i*-diffraction minimum or maximum order on the axis *x* is determined as:

4 $$\begin{aligned} x_i(min)=\pm i.\frac{L\lambda }{b}; \ x_i(max)=\pm \left( i-\frac{1}{2} \right) \frac{L\lambda }{b}, \end{aligned}$$

where $i=1,2,...,k,$ the integers. In single-slit experiments, photon beams show only diffraction patterns following Distribution (1), in particular, the central band $D_m^{dif}$ between the two first minima , i.e. between $x_i=\pm x_1$, reads:

5 $$\begin{aligned} D_m^{dif}=2.L\frac{\lambda }{b_m}. \end{aligned}$$

In a double-slit observation, the interference fringes appear restrictedly within each diffraction pattern with interference $D_m^{int}$. The length of $D_m^{int}$ links with its corresponding diffraction pattern $D_m^{dif}$ as follows:

6 $$\begin{aligned} D_m^{int}\equiv D_m^{dif}-\Delta F. \end{aligned}$$

The number of fringes in the central band is determined as:

7 $$\begin{aligned} n_m=\frac{D_m^{int}}{\Delta F}=2\frac{d}{b_m}-1. \end{aligned}$$

The condition of interference additional minimum and maximum orders in a diffraction pattern are determined as follows:

8 $$\begin{aligned} x_j(min)=\pm \left( j+\frac{1}{2} \right) \Delta F; \ x_j(max)=\pm j.\Delta F, \end{aligned}$$

where $j=0,1,2,...,k$, the integers. Let us recall that the main diffraction minima $x_i(min)$ now serve the missing interference orders.

**Figure 6:**
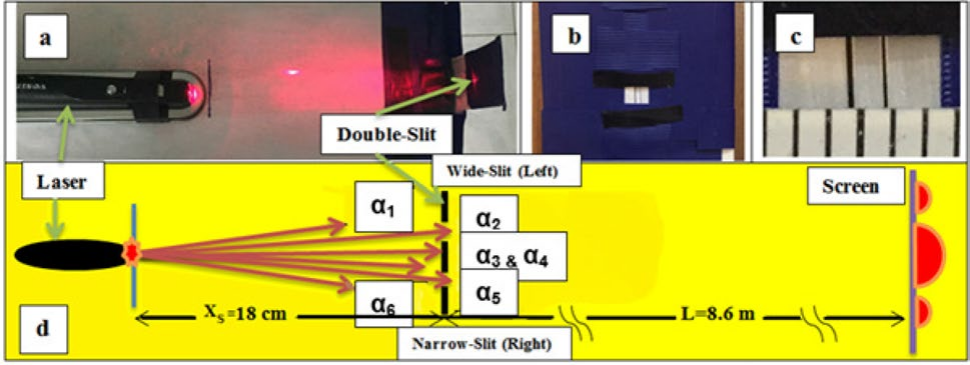
a/ Laser source; b/ Asymmetrical slits and c/ Amplification for measuring their sizes (in mm); d/ Layout of the experiment ($L=8.6$ m, $X_S=0.18$ m) with definition of six horizontal directions of the laser beam at angles $\alpha _1 \div \alpha _6$.

For an asymmetrical double-slit experiment ($b_1>b_2$) assuming that when the laser beam points to a given slit $b_m$, it is expected which way photons pass through, the diffraction pattern then gets a size corresponding to the slit width $b_m$, following Formula (5). Based on the assumption of ontological physical reality, when laser intensity is low enough for self-interference, imitating a condition in experiments with single particles^1,2,5^, the double-slit mixing spectrum consists of two independent interference patterns $D_m^{int}$ of $I_m$-photons, which pass through a given *m*-slit, but not the other slit. Moreover, their interference fringes should overlap in the central band $D_m^{int}$ following the luminance distribution with the minor interference maxima (8) as follows:

9 $$\begin{aligned} I_S^{int}(x)=2.\left[ I_1(x) +I_2(x) \right] . \cos ^2 \left( \frac{\pi . d. x}{L.\lambda }\right) , \end{aligned}$$

where $I_m(x)$ are determined by (1). A minor term $\delta _M(I_1,I_2)\le \frac{1}{\sqrt{2}}\frac{I_1.I_2}{ I_1+I_2}$ (in the vicinity of the main minima) would be added to the major sum (9) to account for possible uncertainty due to multi-photon interference being reminiscent of classical waves. Probably, for a small multi-photon effect, the interference of classical waves leads to a still similar distribution like the ontologically-based summation (9). However, a significant multi-photon contribution will make the two constituent interference patterns now getting strongly mixed, i.e. no more separately independent. Like classical waves, in approaching the center of each pattern, where a violent saturation would often happen, multi-photon interference is getting more and more dominant, which causes an observable deviation from Summation (9). This emphasizes, why a search for the single-photon approximation in vicinities of the main diffraction minima is extremely important. In general, $I_1(x) \ne I_2(x)$ and an inequality of diffraction pattern lengths, e.g. $D_1^{int} < D_2^{int}$ in an asymmetrical double-slit interference would open an opportunity for identification of the path of single photons. Namely, because the missing orders of the two constituent patterns of Summation (9) are not coincident, an interference fringe of a certain pattern has a chance to be recognized in the empty location of a missing order of another pattern, which can reveal the unique path of photons in this fringe. This phenomenon would be a pure experimental which-way identification.

### Experimental set-up

In the present experiment, the narrow slit is designed with a fixed width to form more stable interference fringes. As shown in the layout in Fig. 6 the spacing distance *d* between the two slits made wider ($d\ge 1$mm) allows splitting the central laser direction into two sub-directions at $\alpha _3$ and $\alpha _4$. Consequently, the two fixed slit widths are designed as $b_1=(0.15\pm 0.01)$ mm and $b_2=(0.087\pm 0.010)$ mm, correspondingly, and the distance equal $d=(1.04\pm 0.05)$ mm. We use a digital photo camera for picturing spectra on the screen and use a linear meter for measuring spectrum sizes (in cm).

Figure 6a shows the laser source with the red photon beam ($\lambda =650 \pm 30$) nm while Fig. 6b and c show the diaphragm with asymmetrical slits and their amplification for measuring their widths in mm; The screen with spectrum images located on a distance $L=8.6$ m from the diaphragm satisfies the far-field condition.

A red laser pointer Vesine VP101 is used as the source of monochromatic photons. A non-metallic mask with a rectangular hole constrains the beam size as $S_{Laser}\equiv w$x$h\le (1.5$ × 2.0) ($mm^2$) to meet the sizes of slits with a height $h_{Slit}\approx 3.$ mm and a spacing distance $d\approx 1.$ mm. The laser intensity is $I_{Laser} \le 5.$ mW, however, a relation of slit widths $b_m\le 0.1 w$ makes the effective intensity reduced to $I_m \le 10^{15}$ photons/sec., in particular, $I_m\le 10^{14}$ photons/sec. in the vicinity of the first main minima, which leads roughly to a satisfactory condition of self-interference of a single photon coming in each typical time-window $\Delta t_{Laser}=0.5 \lambda /c \approx 10^{-15}$ sec. of a red laser. The diaphragm with asymmetrical double-slits is made of aluminum foil with asymmetrical slits ($b_1 > b_2$). A ratio R between the two diffraction patterns with $1.5 \le R\le 2.5$ is optimal, otherwise, too different intensities of photons passing the two slits, i.e. $I_1 \gg I_2$ would cause systematic uncertainties.

**Figure 7:**
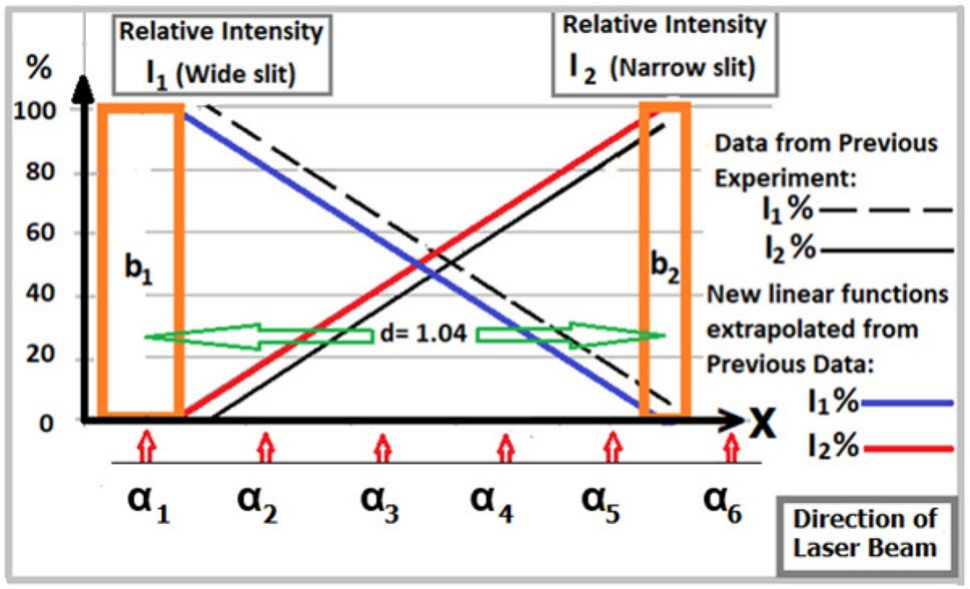
Based on Relative intensities extrapolated from^23^ (the same slit-widths $b_1$, $b_2$) one can determine the relative intensity $I_1/I_2$ in each laser beam direction.

For recording different kinds of spectra, the laser beam is directed to different angles in the horizontal plane, adjusted within an azimuth angle $\alpha \approx 1.1$ milliradian. Adjustment of the laser directions creates different relative integral intensities $I_1/I_2$, leading to different summation luminance distributions following Equation (9). In the results, in each $j-$order in (9) the interference fringes of both components coincide, sharing their certain contribution in a proportion, depending on the corresponding relative quantity $I_1/I_2$. On a distance $X_S=18$ cm from the laser source to the slit diaphragm the beam direction at six angles $\alpha _l$ shifts each step of $\Delta \alpha \approx 0.22$ mrad (or $\Delta x= 0.25$ mm) from the left edge to the right edge within the constraining azimuth $\alpha$, which is calculated as $\alpha _l=(l-1)\Delta \alpha$, were $l=1\div 6$. In Fig. 7 there are six directions shown: i/ Direction at $\alpha _1$ or $\alpha _6$ along the edges of the constraining angle $\alpha$ almost entirely looks in one of the slits, wide or narrow, correspondingly, which expects to create the diffraction patterns $D_m^{dif}$ almost without interference, imitating single slit experiments; ii/ Direction at $\alpha _2$ or $\alpha _5$ looks predominantly in the same slits as does the direction at $\alpha _1$ or $\alpha _6$, shifting a little to the other slit, which creates additional interference fringes within corresponding diffraction patterns $D_m^{int}$; iii/ Directions at $\alpha _3$ and $\alpha _4$ look relatively in the middle between the two slits, with a little adjustment on the left or the right, which create mixing interference fringes of both diffraction patterns $D_m^{int}$ with a moderate difference between $I_1$ and $I_2$. An ISee-scanner then converts 2D images of diffraction patterns to corresponding experimental luminance distributions within statistical errors of $5.\%$ to $10\%$ in the vicinity of the first main minima, serving for reference with their theoretical curves. Other main parameters of the present experiment including the layout remaining almost the same as in^23^ allow using some important outputs of the previous experiment, e.g. the relative integral intensity $I_1/I_2$ depending on the laser beam directions. The larger spacing distance *d* slightly modifies this relative intensity, which can be extrapolated from the old data as presented in Fig. 7.

The latter provides an estimated relative integral intensity in each beam direction for calculation of the corresponding Fraunhofer distribution curve following Summation (9). Before comparison of a theoretical curve with an ISee-spectrum, one would need restoration of an experimental distribution due to the dead-time of the digital photo camera. Following the correction, the experimentally observed rate $I_{obs}$ turns to $I_{true}$ as follows:

10 $$\begin{aligned} I_{true}=\frac{I_{obs}}{1.-\tau _d.I_{obs}}, \end{aligned}$$

where the same dead-time factor $\tau _d\approx 0.012\pm 0.0015$ of the photo camera is applied. A satisfactory correction would be reached in an area with low intensity until an experimental rate $I_{obs}\le 0.6 \tau _d^{-1}$. In principle, an exceed experimental sampling ($N_j>0.6\tau _d^{-1}$) would be corrected by a smaller dead-time factor $\tau _d^N$. However, for convenient comparison, one can use the same $\tau _d=0.012$ in Formula (10) after a renormalization $\tau _d^N=\tau _d/R_N$ and $N_j = I_{obs}R_N$ to meet the requirement. The renormalization factor $R_N$ should be applied to whole experimental samples.
